# Research on Properties of Borocarbide in High Boron Multi-Component Alloy with Different Mo Concentrations

**DOI:** 10.3390/ma14133709

**Published:** 2021-07-02

**Authors:** Xiangyi Ren, Lihong Han, Hanguang Fu, Jianjun Wang

**Affiliations:** 1 State Key Laboratory of Performance and Structural Safety for Petroleum Tubular Goods and Equipment Materials, CNPC Tubular Goods Research Institute, Xi’an 710077, China; 2 Research Institute of Advanced Materials Processing Technology, School of Materials Science and Engineering, Beijing University of Technology, Beijing 100124, China; hgfu@bjut.edu.cn (H.F.); wangjianjun005@cnpc.com.cn (J.W.)

**Keywords:** high-boron multi-component alloy, borocarbide, molybdenum, first-principles calculation, nano-indentation

## Abstract

In this work, the microstructure, alloying element distribution, and borocarbide mechanical properties of high-boron multi-component alloy with Fe-2.0 wt.%B-0.4 wt.%C-6.0 wt.%Cr-*x* wt.%Mo-1.0%Al-1.0 wt.%Si-1.0 wt.%V-0.5 wt.%Mn (*x* = 0.0, 2.0, 4.0, 6.0, 8.0) are investigated. The theoretical calculation results and experiments indicate that the microstructure of high-boron multi-component alloy consists of ferrite, pearlite as a matrix and borocarbide as a hard phase. As a creative consideration, through the use of first-principles calculations, the comprehensive properties of borocarbide with different molybdenum concentrations have been predicted. The calculations of energy, state density, electron density and elastic constant of Fe_2_B crystal cell reveal that substitution of the molybdenum atom in the Fe_2_B crystal cell can remarkably improve its thermodynamic stability, bond strength, and covalent trend. For verifying the accuracy of this theoretical calculation, nano-indentation testing is carried out, the results of which indicate that the actual properties of borocarbide present favorable consistency with the theoretical calculations.

## 1. Introduction

As a kind of cost-effective resource in China, boron has been widely investigated in the past few years. Researchers indicated that a matrix of steel with a slight boron solution has excellent properties, such as hardenability and mechanical strength. When boron content further increases, boride with a high hardness and thermal stability is formed in the microstructure [1,2,3,4,5,6,7,8]. For wear-resistant ferrous alloys, through addition of boron instead of alloying elements, such as V, W, Ti, etc. [9,10,11,12,13,14,15], production costs can be reduced and the manufacturing process can be simplified. High-boron multi-component alloy is a kind of new-type wear-resistant material. The hardness of borocarbide in this alloy is 1500–1700 HV. However, borocarbide in general high-boron multi-component is unable to bear abrasion because of its poor toughness. [14,16].

Molybdenum is a kind of widely used alloying element in alloy steel, which possesses the effect of segregation elimination, carbide formation promotion, and improves hardenability and toughness. For boron-added ferrous alloy, it is significant for improving its toughness. Most molybdenum in high-boron multi-component alloy exists in borocarbide in the form of a solid-solution, which possesses the effects of improving the morphology and toughness [17].

According to previous investigations, the basic physical properties of solid materials depend on their internal electronic conditions [18,19,20,21,22]. Thus, the macro properties of materials can be theoretically predicted through the acquisition of their electronic structures, energy, and other information from the crystal. In recent years, with the development of servers, and other relevant equipment, first-principles calculations, which are based on the density functional theory (DFT), have been widely used in the calculation of electronic structure, stability, and the mechanical properties of condensed state materials [23,24,25,26,27,28,29]. According to the information above, it is inferred that the properties of borocarbide in a high-boron multi-component alloy can be predicted using first-principles calculations.

This work systematically investigates the microstructure, alloying element distribution, and mechanical properties of high-boron multi-component alloy with compositions of Fe-2.0 wt.% B-0.4 wt.% C-6.0 wt.% Cr-*x* wt.% Mo-2.0% Al-1.0 wt.% Si-1.0 wt.% V-0.5 wt.% Mn (*x* = 0.0, 2.0, 4.0, 6.0, 8.0). Meanwhile, with the help of first-principles calculations of the borocarbide lattice with various Mo atom additions, the effect of molybdenum on the morphology and mechanical properties of borocarbide is studied.

## 2. Experimental Procedure

### 2.1. Materials and Specimens

Pure industrial aluminum ferrous alloys, including boron, carbon, molybdenum, chrome, manganese, silicon, and vanadium, respectively, were used as raw materials for the manufacturing of high-boron multi-component alloy. The above-mentioned materials were smelted in a 1600 °C induction furnace under air atmosphere. Liquid steel was poured into a pre-heated Y-block mold which is made of dried sand at 1500–1550 °C. Specimens for metallurgical analyses were 15-mm cubes cut from the center of the ingot.

### 2.2. Characterization

X-ray fluorescence analyses (XRF) were used to determine the chemical composition of the studied alloys, the results of which are shown in Table 1. An optical microscope (OM), X-ray diffraction (XRD), scanning electron microscope (SEM), and electron probe microanalyzer (EPMA) were used to characterize the microstructures of the metallurgical specimens. All of the metallurgical specimens were mechanically polished and then etched by 5% nital. Polished specimens for volume fraction calculations needed to be dyed using Carlin corrosive.

The volume fraction reflects the amount of borocarbide [30,31], which can be directly calculated by the image analyzing software, ImageJ. According to the stereological formula (1), volume fraction *V_V_* could be replaced by area fraction *A_A_* in the microstructure image.

*V_V_* = *A_A_* (1)

The TI950 nano-indentation tester was used to investigate the hardness, elasticity, and plasticity of borocarbide in the studied alloys with different Mo contents. The pit caused from the plastic deformation on one of the phases in the alloy, caused by a Berkovich indenter, could be used to analyze the relationship between load and deformation, the hardness, elastic modulus, the plastic deformation resistance, and other parameters of this phase can also be obtained. The experimental parameters of this work are shown in Table 2.

## 3. Results and Discussion

### 3.1. Microstructure of High Boron Multi-Component Alloy with Different Molybdenum Contents

**Figure 1:**
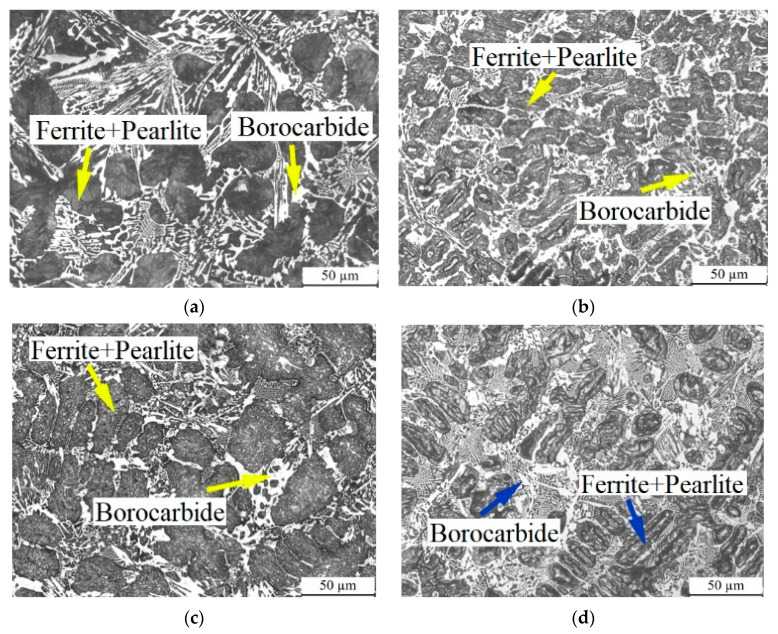

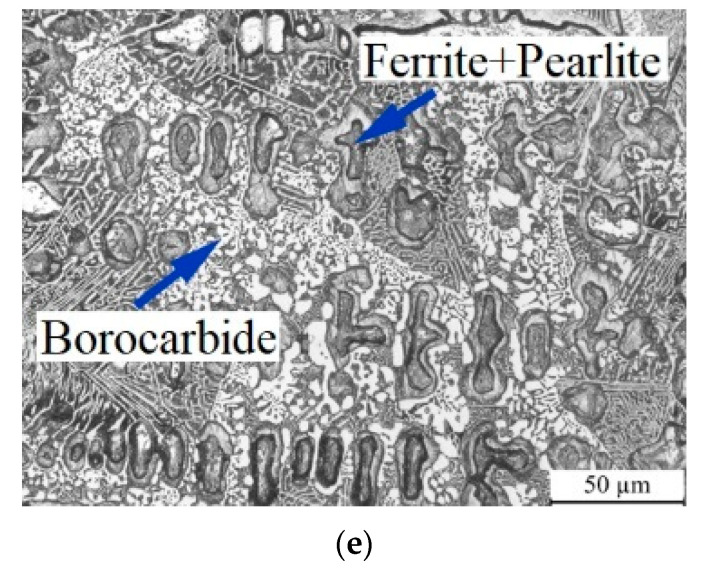
Morphology of high-boron multi-component alloy with different Mo contents: (**a**) 0.0 wt.%, (**b**) 2.0 wt.%, (**c**) 4.0 wt.%, (**d**) 4.0 wt.%, (**e**) 8.0 wt.%.

Figure 1 shows the microstructure of high-boron multi-component alloy with different molybdenum concentrations. It was observed that the alloy consisted of ferrite and pearlite (dark area) and a light reticular structure. The reticular structure size in sample M0 was quite large. With the increase in molybdenum content, the size of the reticular structure was reduced gradually, as shown in Figure 1b–e. In addition, some type of extremely fine reticular structures appeared and increased with an increase in Mo content. When the Mo content reached 6 wt.%, it could be seen that quantity of tiny reticular structures was higher than that of the normal reticular structures.

**Figure 2:**
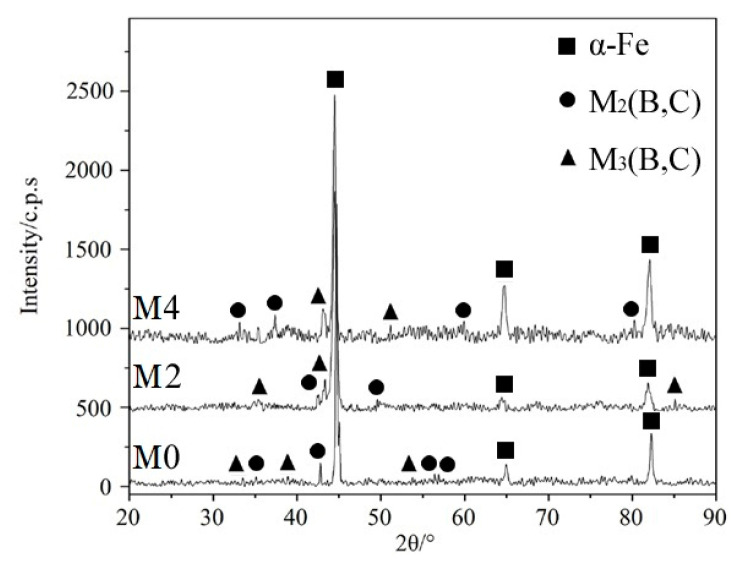
XRD patterns of alloys with the Mo contents of 0.0 wt.%, 4.0 wt.% and 8.0 wt.%.

For certifying the phase types of the high-boron multi-component alloy, XRD analyses of alloy M0, M2 and M4 were carried out, and results are shown in Figure 2. It was observed that α-Fe as matrix and borocarbide M_2_(B,C), M_3_(B,C) (M = Fe, Cr, Mo, V, Mn) were detected in the tested alloys. M_2_(B,C) was the resultant of eutectic reaction, which existed as reticular borocarbide in the microstructure. After solidification, boron atoms and other metal atoms diffused into cementite in the pearlite, resulting in the formation of boron–cementite M_3_(B,C). In addition, according to the difference in the three patterns, it was inferred that the variation in Mo content had no effect on the phase types of high-boron multi-component alloy.

**Figure 3:**
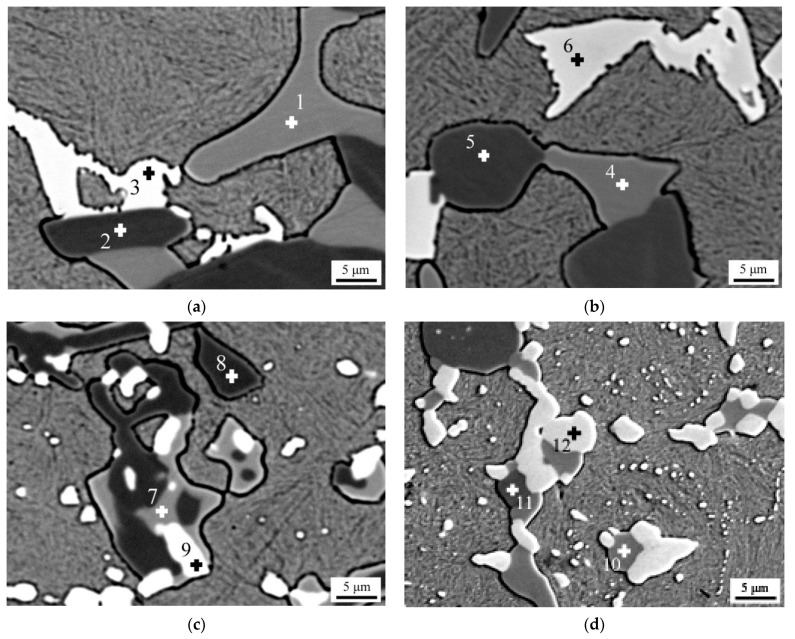
SEM BSE morphologies of high-boron multi-component alloy with various Mo contents: (**a**) 2.0 wt.%, (**b**) 4.0 wt.%, (**c**) 6.0 wt.%, (**d**) 8.0 wt.%.

The SEM BSE observations were carried out for investigating the effect of Mo content on the element distribution of high-boron multi-component alloy, the results of which are shown in Figure 3. The SEM BSE morphology reflects the inhomogeneous distribution of elements in borocarbide. Three kinds of borocarbide with different compositions were observed. Table 3 shows the EPMA point scanning results of the studied alloys with different Mo contents. No variations in Mo content were detected in borocarbide at points 1, 4, 7, and 10. In this kind of borocarbide, Mo concentration increased only when the Mo content was greater than 4.0 wt.%. Borocarbide at points 2, 5, 8, and 11 possessed similar compositions and a relatively high Cr content, in which the Mo concentration remained constant when the Mo content in the alloy changed. The composition of Mo-rich borocarbide is reflected at points 3, 6, 9, and 12. Moreover, the Mo concentration in Mo-rich borocarbide increased when the Mo content in alloy reached 4.0 wt.%.

**Figure 4:**
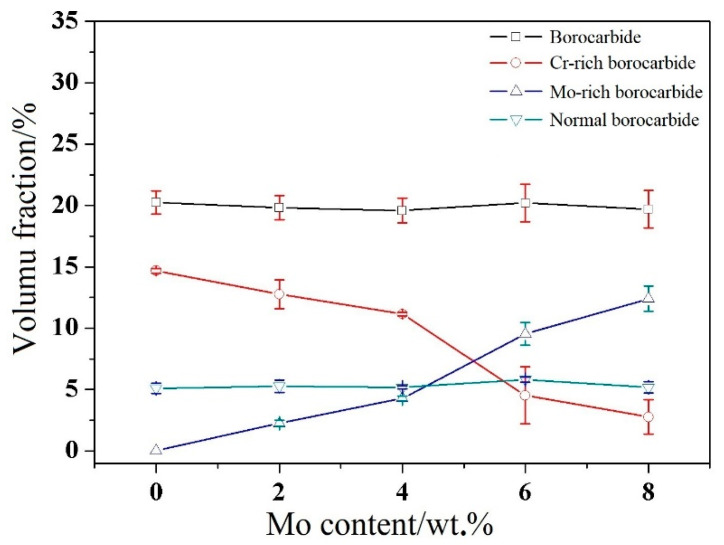
Borocarbide volume fraction calculation results of high-boron multi-component alloy with different Mo contents.

To certify whether the Mo content has an effect on borocarbide quantity, a borocarbide volume fraction calculation was carried out, the results of which are shown in Figure 4. It can be seen that the variations in Mo content had no effect on the entire quantity of borocarbide. The volume fraction of borocarbide was approximately 20% in each alloy. However, the quantity of borocarbide with different compositions possess obvious and regular changes. With an increase in Mo content, the volume fraction of the Mo-rich borocarbide increases, but Cr-rich borocarbide decreases. These calculated results, together with the observed morphology results of the studied alloys revealed that the main type of borocarbide is Cr-rich borocarbide when the Mo content is relatively low, which changes into Mo-rich borocarbide when the Mo content is greater than 4 wt.%.

### 3.2. First-Principles Calculation of Borocarbide with Different Mo Concentrations in High-Boron Multi-Component Alloy

From the previous results, molybdenum exists in borocarbide in the form of a solid solution. According to crystallographic principles, when one or more atoms are substituted by allochthonous atoms in a crystal cell, the shape, electronic environment, and other parameters of this cell will be changed. Thus, it is inferred that borocarbide with different Mo contents possesses various quantities of Mo atom substitutions in the crystal cell. This work uses the CASTEP model of material calculation software, Materials Studio, in which the crystal cell of M_2_B (M = Fe, Mo) with the substitutions of 0, 1, 2 and 3 Mo atoms, respectively, can be created. These cells created by software can be used for structural and energetic information calculations and mechanical property simulations. The calculating results can be applied for revealing the effects of Mo substitution in a M_2_B cell on the general theoretical properties of borocarbide.

Before creating the M_2_B crystal cell, information of the space group, lattice type, and parameters of the cell needed to be obtained from the PDF card. Details of the information are shown in Table 4. To ensure the conforming of the stimulated results and the actual situation, and considering the lattice distortion caused by Mo atom substitution, carrying out the geometric optimization after crystal cell creation was necessary. The set parameters of the geometric optimization are shown in Table 5, which are based on the lattice parameters shown in Table 4. In addition, establishment of the k-point range should be consistent with the corresponding reciprocal space of the lattice parameter. The crystal cell after geometric optimization can be used for the calculation of population analyses, state density, energy information, and elastic constant.

**Figure 5:**
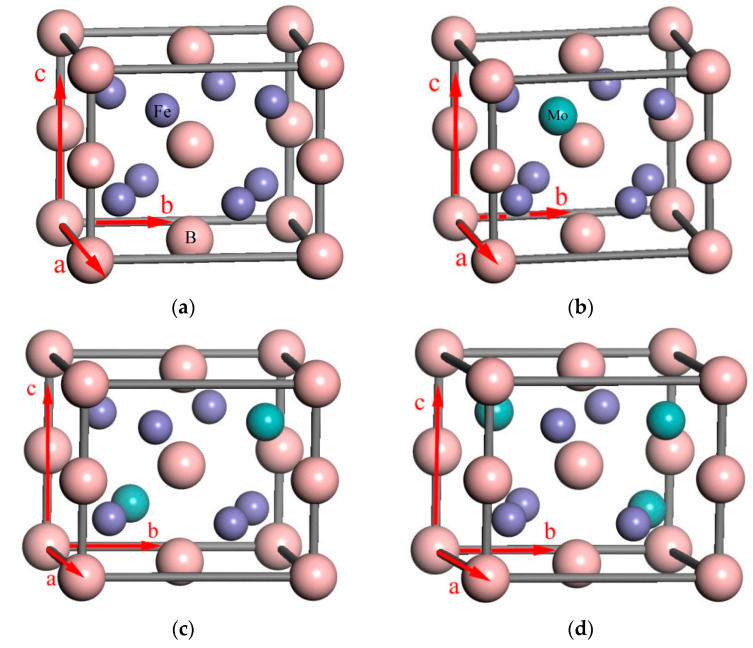
Lattice structure of M_2_B with different numbers of Mo atoms: (**a**) Fe_8_B_4_, (**b**) Fe_7_MoB_4_, (**c**) Fe_6_Mo_2_B_4_, (**d**) Fe_5_Mo_3_B_4_.

Figure 5 shows the ball–stick model of the M_2_B crystal cell with different numbers of Mo atom substitutions, created using Materials Studio software. It was inferred that the molecular formula of these cells were Fe_8_B_4_, Fe_7_MoB_4_, Fe_6_Mo_2_B_4_ and Fe_5_Mo_3_B_4_, respectively.

Table 6 shows the geometric optimization results of the studied cells, which accurately reflect the structural information and distortion degrees of these cells. From these data, cells with Mo substitutions possess smaller values of a and b than those without Mo substitutions. Values of a and b change slightly with a variation in Mo atoms in the cells. However, the value of lattice parameter c increases with an increase in Mo atom numbers in cells. The Fe atom, the radius of which is 0.127 nm, contains 26 extranuclear electrons. The Mo atom contains 42 extranuclear electron, and its radius is 0.140 nm. When the Fe atom in the crystal cell is replaced by a Mo atom, part of the cell swells because of the substitution of the Mo atom with larger radius. Moreover, the atomic force of the Mo atom with B and Fe atoms increases. All of the variation mentioned above cause a change in the Mo-included crystal cell, reflected in Table 6.

The energy calculation results of the M_2_B crystal cells with different contents of Mo atoms are shown in Table 7. From the point of view of crystal cell volume, cells with one and two Mo atoms possessed the smallest and largest values, respectively. When the crystal cell contained one or three Mo atoms, the distribution of Mo atoms in cell was inhomogeneous. For balancing the internal atomic force, half of the cell without Mo atoms reduced. The degree of this reduction was larger than the swelling degree of the part with more Mo atoms. Thus, cell Fe_7_MoB_4_ presented the smallest volume. Simultaneously, the volume of cell Fe_5_Mo_3_B_4_ was smaller than that of cell Fe_6_Mo_2_B_4_. When two Mo atoms possessed homogeneous distributions in a cell, the whole cell swelled because of the increase in the atom radius and the atomic force. All of the cells possessed negative energy values, which indicated that they can stably exist in the microstructure. Moreover, the energy of the cells decreased with the increase in Mo atom numbers. Thus it was inferred that the Mo atom can improve the properties, especially high temperature stability, of borocarbide by improving the thermodynamic stability of the crystal cell. Reference [32] suggests that the brittleness of Fe_2_B is caused by the low B–B bond energy along the [2] crystal orientation. According to the results of B–B bond length in Table 7, when 1 or 3 Mo atoms are in a cell, the length of B–B bond in the [2] direction reduced. When the cell contained 2 Mo atoms, the length increased. Moreover, the length of B–M bond (M = Mo, Fe) was lower than that of the B–Fe bond. Decreasing the bond length results in an increasing bond energy. Thus, it was theoretically revealed that the substitution of a Mo atom in Fe_2_B crystal cell can effectively reinforce the B–B and B–M bonds, so that the brittleness of borocarbide can be improved.

**Figure 6:**
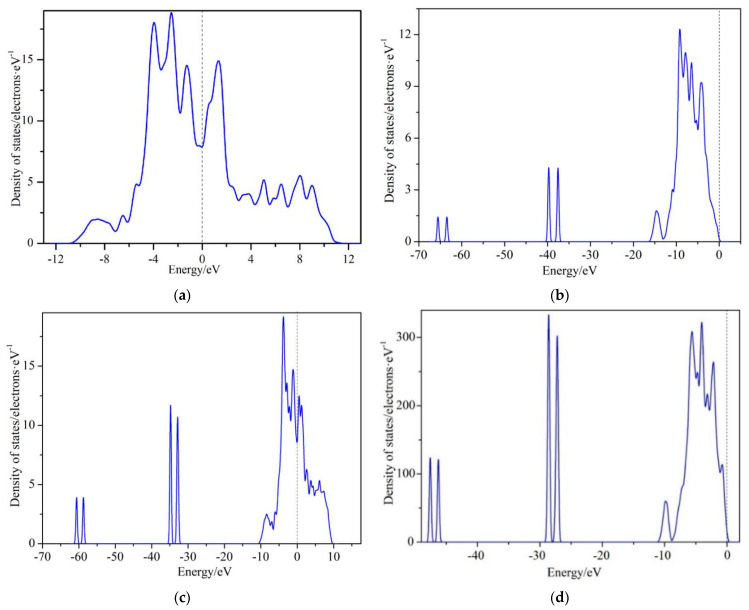
State density of M_2_B with different numbers of Mo atoms: (**a**) Fe_8_B_4_, (**b**) Fe_7_MoB_4_, (**c**) Fe_6_Mo_2_B_4_, (**d**) Fe_5_Mo_3_B_4_.

The calculation of the state density for cells with different Mo atom concentrations can reflect the stability of extranuclear electrons and interactions of the atoms. The closer to zero the state density at the Fermi surface is, the more stable the extranuclear electrons are. Figure 6 shows the calculated results of the state density of the M_2_B crystal cell. It was observed that cells with an asymmetrical structure, such as Fe_7_MoB_4_ and Fe_5_Mo_3_B_4_, possess quite low values of state density at the Fermi surface. However, the state density of cell Fe_6_Mo_2_B_4_ at the Fermi surface is higher than that of cell Fe_8_B_4_. Generally, the lower density of the state at the Fermi surface (0 on the abscissa), the stronger the trend of the covalent bond the cell possesses. The strength of a covalent bond is much higher than other types of bonds. Consequently, these phenomena reveal that Mo concentrations in the cell can improve the bond strength. It is further proved that Mo concentrations in Fe_2_B crystal cell can improve its properties by increasing the bond strength.

**Figure 7:**
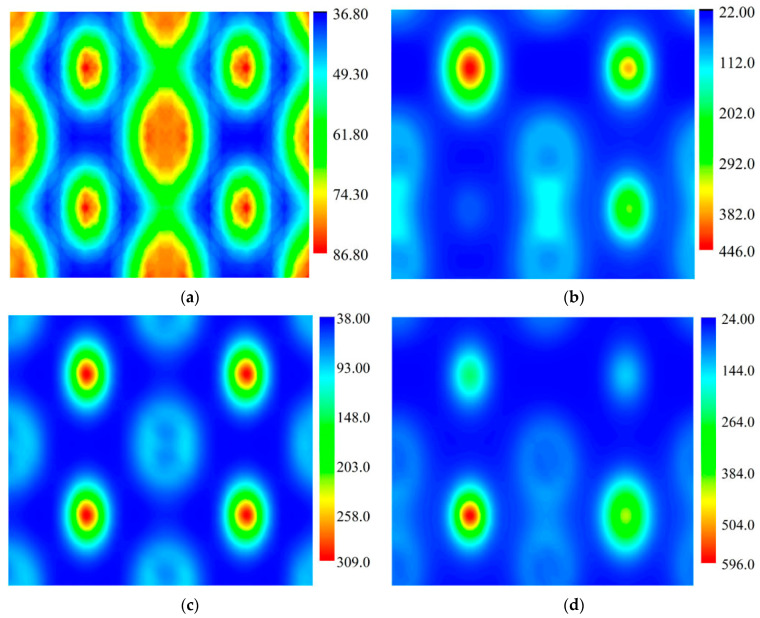
Total electron density on the (110) crystal plane of M_2_B with different numbers of Mo atoms: (**a**) Fe_8_B_4_, (**b**) Fe_7_MoB_4_, (**c**) Fe_6_MO_2_B_4_, (**d**) Fe_5_Mo_3_B_4_.

As mentioned before, the state density reflects the interacting type of electrons in the crystal cell. For revealing the degree of this interaction, calculations of the electron density on the (110) crystal plane was carried out, the results of which are presented in Figure 7. When no Mo atom is in the cell, the level of the electron density is relatively low. In general, the Fe_2_B crystal cell, bond along the [2] orientation is weakest. It is worth noting that the electron density along the [2] orientation is only approximately 60 e/Å^2^. When one or more Fe atoms are substituted by Mo atoms, the level of electron density in the whole cell increases remarkably. Moreover, the density of boron atoms along the [2] orientation also increases obviously. Cell Fe_5_Mo_3_B_4_ presents the highest electron density (approximately 140 e/Å^2^). Consequently, the bond strength especially the B–B bond with a lower strength along the [2] orientation can be reinforced with the substitution of Mo atoms. In conclusion, substitution of Mo atom in the Fe_2_B crystal cell can effectively improve thermodynamic stability and bond strength by increasing the electron stability, density, and shortening the B–B bond length.

The utilization of first-principles calculations can, not only predict the property of a crystal cell using structural and energy calculations, but can also calculate the mechanical properties directly. Results of elastic constant calculations of M_2_B crystal cells with different Mo atom concentrations are presented in Table 8. The ratio of the bulk modulus and the shearing modulus can reflect the toughness of the material. The higher value of this ratio, the better the toughness of the material [33,34]. When the cell contains one Mo atom, the toughness rises slightly. However, other parameters are reduced. When two Mo atoms exist in a cell, all of the parameters increase. In cells with three Mo atoms, the toughness increases but the elastic modulus decreases. Together with the structural and energy calculation results, it could be revealed that the substitution of the Mo atom in the Fe_2_B crystal cell improves the thermodynamic stability, bond strength, and mechanical properties remarkably. Consequently, the improvement in the general properties of borocarbide in high-boron multi-component alloy based on molybdenum concentration can be theoretically proved.

### 3.3. Experimental Testing of Mechanical Properties of Borocarbide with Different Mo Concentrations in High-Boron Multi-Component Alloy

**Figure 8:**
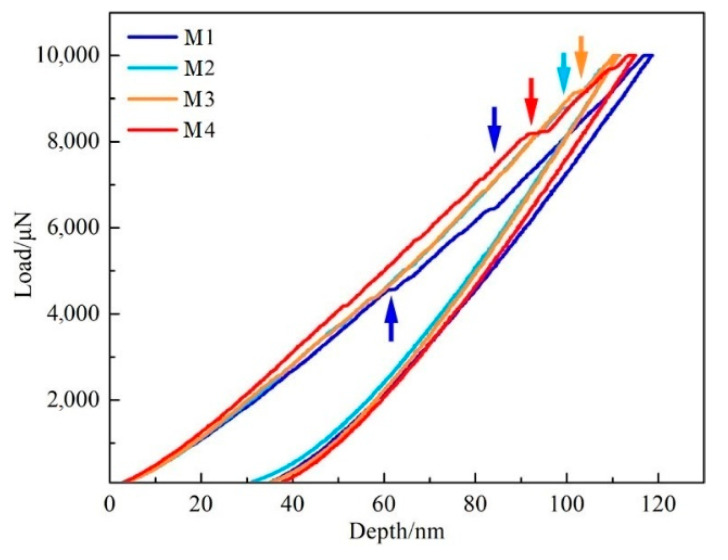
Depth–load curve of Mo-rich borocarbide with different Mo contents.

For verifying whether the results of the predicted properties using first-principles calculations are in accordance with the actual properties of borocarbide, it is necessary to investigate the mechanical properties of borocarbide through experiments. According to the previous results, the size of Mo-rich borocarbide is quite small, with which regular hardness testing cannot be carried out. Consequently, property testing of Mo-rich borocarbide needs to be implemented using the nano-indentation method. Through this testing method, the corresponding relationships of borocarbide deformation and load on the borocarbide surface during a loading and unloading process can be obtained. This relationship can be used to reveal the hardness, elasticity, and toughness of borocarbide. Figure 8 shows the load–depth curve of Mo-rich borocarbide for samples M1, M2, M3 and M4. The curves on the left side reflect the loading process. The right side of the curves reflects the unloading process. Different samples possess various extents of deformation, which indicates that these samples have different hardnesses. Sample M2 presents the highest value. The gradient of the curves reflects the elastic modulus of borocarbide. A high modulus corresponds to a high curve gradient. Through the elastic modulus calculation, it is indicated that the elastic modulus of borocarbide increases with proper Mo addition. The loading curve and unloading curve do not coincide, which indicates that all of the samples remain plastic deformation after the unloading process. With the increase in Mo content in the alloy, the plasticity of borocarbide decreases first and then increases. Sample M2 presents the best plastic deformation resistance, because it shows the lowest pit depth after the indentation process.

Table 9 shows the measurement results of hardness and elastic modulus of Mo-rich borocarbide in the studied alloys, which correspond to the trends in Figure 8. In Table 9, the hardness of the cells undulates in a small range, which indicates that the variation in Mo content has no obvious effect on the improving the hardness of the borocarbide. The elastic modulus increases with the increase in Mo content in the cell. Generally, materials with a high elastic modulus reflect good deformation resistance. Thus, it is inferred that Mo addition in the cell effectively improves its strength. The arrows in Figure 8 point the steps in each curve. These steps reflect the critical points of elastic and plastic deformation of borocarbide. The higher the load that corresponding to the step, the better the plastic deformation resistance of borocabide. It is worth noting that more than one step appears in the loading curve of alloy M1, which indicates that alloy M1 has no obvious critical point of elastic and plastic deformation. In the rest of the alloys, only one step appears in each curve. Moreover, when the Mo content is greater than 6 wt.%, plastic deformation resistance of the alloy reduces. In sum, proper Mo addition in borocarbide can remarkably improve its hardness, elastic modulus, and plastic deformation resistance.

## 4. Conclusions

(1) The microstructure of high-boron multi-component alloy consists of ferrite, pearlite and borocarbide. The alloying element in borocarbide possesses an inhomogeneous distribution, resulting in the appearance of Mo-rich, Cr-rich, and normal borocarbide. With the increase of Mo content, proportion of Mo-rich borocarbide gradually rises.
(2) The first-principles calculations executed using Materials Studio software are used to theoretically predict the comprehensive property of borocarbide. Through the calculation of the energy, state density, electron density and elastic constant of crystal cell, it is theoretically revealed that substitution of the Mo atom in the Fe_2_B crystal cell can effectively promote the thermodynamic stability, elastic modulus, bond strength and covalent trend. Consequently, the hardness and toughness of borocarbide are reinforced obviously.
(3) Through the utilization of nano-indentation testing, the theoretical prediction of the mechanical properties of borocarbide using first-principles calculations is experimentally proved. With the addition of Mo to borocarbide, hardness, elastic modulus and plastic deformation resistance were improved remarkably.

## Figures and Tables

**Table 1:** Chemical composition of the studied alloys (wt.%).

Samples	B	C	Cr	Mo	Al	Si	V	Mn	Fe
M0	1.8	0.4	5.5	0.0	0.7	1.0	0.9	0.6	Bal.
M1	1.8	0.4	5.5	2.1	0.8	1.0	1.0	0.6	Bal.
M2	2.0	0.4	5.9	3.9	0.9	0.9	0.9	0.5	Bal.
M3	1.8	0.4	5.4	6.2	0.7	1.0	1.0	0.6	Bal.
M4	1.8	0.4	5.4	8.3	0.7	1.0	1.0	0.6	Bal.

**Table 2:** Experimental parameter of nano-indentation.

Parameter Types	Value
Max load	10 mN
Loading time	5 s
Loading rate	2 mN/s

**Table 3:** EPMA point scanning results of high-boron multi-component alloy with different Mo contents (wt.%).

Testing Point	B	C	Cr	Mo	Al	Si	V	Mn	Fe
1	3.55	0.45	9.10	4.19	0.02	0.00	1.80	0.79	80.1
2	9.83	0.64	15.10	1.38	0.00	0.00	2.05	0.76	70.24
3	9.04	0.42	11.42	41.57	0.01	0.00	8.79	0.38	28.37
4	4.12	0.78	7.95	3.82	0.03	0.01	1.41	0.86	81.02
5	7.07	0.66	13.38	1.87	0.02	0.02	2.07	0.93	73.98
6	8.96	0.62	10.67	43.51	0.02	0.02	7.42	0.41	28.37
7	2.28	0.56	10.20	4.93	0.01	0.01	1.39	0.77	79.85
8	8.58	0.10	16.14	1.92	0.03	0.01	1.67	0.77	70.78
9	4.53	0.80	11.99	48.54	0.00	0.02	6.71	0.44	26.97
10	6.35	0.89	8.88	5.10	0.04	0.03	1.02	0.16	77.53
11	6.87	0.60	12.84	1.44	0.00	0.02	0.99	0.65	76.59
12	6.84	0.67	11.40	51.33	0.00	0.01	5.80	0.35	23.60

**Table 4:** Lattice parameters of M_2_B crystal cell.

Parameter	Type
Space group	I-4/mcm
Lattice	Tetragonal
Lattice constant a	0.511 nm
Lattice constant c	0.424 nm

**Table 5:** Parameter settings of geometric optimization in the CASTEP model of Materials Studio.

Parameter	Setting Option
Optimizing mode	BFGS
Optimizing method	GGA-PBE
Cutting energy	300 Ev
SCF convergence energy	10^−5^ eV per atom
k-point range	5 × 5 × 6
Pseudo-potential type	Ultrasoft

**Table 6:** Results of geometric optimization of M_2_B crystal cells with different Mo atom concentration.

Crystal Cells	Lattice Parameters/nm		
	a	b	c
Fe_8_B_4_	0.5880	0.5746	0.4249
Fe_7_MoB_4_	0.5490	0.5501	0.4694
Fe_6_Mo_2_B_4_	0.5665	0.5667	0.4721
Fe_5_Mo_3_B_4_	0.5558	0.5561	0.4737

**Table 7:** Results of energy calculation of M_2_B crystal cells with different Mo atom concentrations.

Crystal Cells	Cell Volume/nm^3^	Cell Energy/eV	B-B Bond Length/nm	B-M Bond Length/nm	M-M Bond Length/nm
Fe_8_B_4_	14.356	−7217.12	0.2112	0.2428	0.2715
Fe_7_MoB_4_	14.176	−8291.65	0.1739	0.2373	0.2731
Fe_6_Mo_2_B_4_	15.156	−9362.59	0.2359	0.2397	0.2822
Fe_5_Mo_3_B_4_	14.641	−10,436.52	0.1817	0.2395	0.2780

**Table 8:** Results of elastic constant calculation of M_2_B crystal cells with different Mo atom concentrations.

Crystal Cells	Bulk Modulus *B*	Shearing Modulus *G*	Elastic Modulus *E*	Poisson Ratio ν	*B/G*
Fe_8_B_4_	124.84	57.48	149.50	0.30	2.17
Fe_7_MoB_4_	110.2	42.86	122.35	0.43	2.57
Fe_6_Mo_2_B_4_	197.40	73.61	214.82	0.46	2.68
Fe_5_Mo_3_B_4_	191.36	78.65	207.52	0.32	2.43

**Table 9:** Results of elastic constant calculations of M_2_B crystal cells with different Mo atom concentrations.

Samples	Mechanical Properties	
	Hardness *H*/GPa	Elastic Modulus *E*/GPa
M1	20.28	209.19
M2	24.01	222.02
M3	20.08	221.53
M4	19.93	244.77

## Data Availability

This study did not report any data online.
